# Experimental Mouse Model of Lumbar Ligamentum Flavum Hypertrophy

**DOI:** 10.1371/journal.pone.0169717

**Published:** 2017-01-06

**Authors:** Takeyuki Saito, Kazuya Yokota, Kazu Kobayakawa, Masamitsu Hara, Kensuke Kubota, Katsumi Harimaya, Kenichi Kawaguchi, Mitsumasa Hayashida, Yoshihiro Matsumoto, Toshio Doi, Keiichiro Shiba, Yasuharu Nakashima, Seiji Okada

**Affiliations:** 1 Department of Advanced Medical Initiatives, Graduate School of Medical Sciences, Kyushu University, Fukuoka, Japan; 2 Department of Orthopaedic Surgery, Graduate School of Medical Sciences, Kyushu University, Fukuoka, Japan; 3 Department of Orthopaedic Surgery, Spinal Injuries Center, Fukuoka, Japan; 4 Department of Orthopaedic Surgery, Kyushu University Beppu Hospital, Oita, Japan; Rush University Medical Center, UNITED STATES

## Abstract

Lumbar spinal canal stenosis (LSCS) is one of the most common spinal disorders in elderly people, with the number of LSCS patients increasing due to the aging of the population. The ligamentum flavum (LF) is a spinal ligament located in the interior of the vertebral canal, and hypertrophy of the LF, which causes the direct compression of the nerve roots and/or cauda equine, is a major cause of LSCS. Although there have been previous studies on LF hypertrophy, its pathomechanism remains unclear. The purpose of this study is to establish a relevant mouse model of LF hypertrophy and to examine disease-related factors. First, we focused on mechanical stress and developed a loading device for applying consecutive mechanical flexion-extension stress to the mouse LF. After 12 weeks of mechanical stress loading, we found that the LF thickness in the stress group was significantly increased in comparison to the control group. In addition, there were significant increases in the area of collagen fibers, the number of LF cells, and the gene expression of several fibrosis-related factors. However, in this mecnanical stress model, there was no macrophage infiltration, angiogenesis, or increase in the expression of transforming growth factor-β1 (TGF-β1), which are characteristic features of LF hypertrophy in LSCS patients. We therefore examined the influence of infiltrating macrophages on LF hypertrophy. After inducing macrophage infiltration by micro-injury to the mouse LF, we found excessive collagen synthesis in the injured site with the increased TGF-β1 expression at 2 weeks after injury, and further confirmed LF hypertrophy at 6 weeks after injury. Our findings demonstrate that mechanical stress is a causative factor for LF hypertrophy and strongly suggest the importance of macrophage infiltration in the progression of LF hypertrophy via the stimulation of collagen production.

## Introduction

Lumbar spinal canal stenosis (LSCS) is a common spinal disorder in elder people. There are approximately 250,000 to 500,000 LSCS patients in the United States, with the number increasing due to the aging of the population [1]. LSCS causes lower back pain, leg pain, and claudication, leading to severe disability in the activities of daily living [2]. These symptoms primarily result from the hypertrophy of the ligamentum flavum (LF). The LF is a spinal ligament that covers most of the posterior and lateral parts of the spinal canal. Thus, hypertrophy of LF directly compresses the nerve roots and/or cauda equine, resulting in spinal disorders [3]. Although there have been several studies about LF hypertrophy, its pathomechanism remains unclear.

In previous studies, only human samples have been used due to the lack of a relevant animal model. A limitation of these studies was the difficulty in observing the gradual process of LF hypertrophy because the hypertrophied LF obtained from LSCS patients already showed advanced histological changes; the loss of elastic fibers and an excessive accumulation of collagen fibers [4,5]. Molecular expression changes were also observed in human hypertrophied LF, such as transforming growth factor-β1 (TGF-β1) [6], connective-tissue growth factor (CTGF) [7] and platelet-derived growth factor (PDGF) [8], which are related to collagen production [9–11]. However, whether such factors are causative or merely a consequence of LF hypertrophy remains unknown. Therefore, basic research using an experimental animal model is necessary to elucidate its etiology.

Various possible factors for LF hypertrophy have been considered, such as age, activity level, genetic components, and mechanical stress [12–14]. Among them, we focused on mechanical stress, which has been considered an influencing factor in previous studies. Indeed, Fukuyama et al. reported that the LF thickness of lumbar degenerative instability patients was larger than that of non-instability patients [12]. Furthermore, mechanical stretching force was reported to increase collagen synthesis in cultured human LF cells [15]. However, there are no *in vivo* studies directly demonstrating that mechanical stress induces LF hypertrophy.

In this study, we established a LF hypertrophy mouse model using a novel loading device and examined the influence of consecutive mechanical stress on LF hypertrophy. In addition, we induced macrophage infiltration into the mouse LF by applying micro-injury and examined the pathological role of macrophages in LF hypertrophy.

## Materials and Methods

### Animals

Eight-week-old female C57BL/6 wild-type mice and CAG-EGFP transgenic mice were used in this study (Japan SLC, Shizuoka, Japan). All mice were housed in a temperature- and humidity-controlled environment with a 12 h light–dark cycle. In all animal experiments, the mice were anesthetized intraperitoneally with an anesthetic mixture (medetomidine 0.3 mg/kg, midazolam 4 mg/kg, and butorphanol 5 mg/kg) every hour in consideration of the anesthetic duration [16]. The animal protocol was approved by the Committee of Ethics on Animal Experiment in Faculty of Medicine, Kyushu University (A-27-220-0) in accordance with the Guidelines for Animal Experimentation. All efforts were made to reduce the number of animals used and to minimize their suffering.

### Histological analysis

After the mice were transcardially fixed with 4% paraformaldehyde, the lumbar spine was removed and immersed in the same fixative. The spine was decalcified in ethylenediaminetetraacetic acid solution and dehydrated in sucrose solution. The sample was then embedded into OCT compound, frozen in liquid nitrogen, and cut into 10-μm sections on a cryostat. The sections were subjected to hematoxylin-eosin (HE) and Elastica-van Gieson (EVG) staining. For immunostaining, the sections were stained with primary antibodies against Iba1 (1:200; macrophage marker; Wako, Osaka, Japan), laminin (1:200; basement membrane marker; Sigma-Aldrich, Saint Louise, MO), collagen type 1 alpha 1 (COL1A1; 1:200; Sigma-Aldrich), and 5-bromo-20-deoxyuridine (BrdU; 1:200; proliferating cell marker; Abcam, Cambridge, UK). Then, the sections were incubated with Alexa Fluor-conjugated secondary antibodies (1:200; Invitrogen, Carlsbad, USA). Nuclear counterstaining was performed using Hoechst 33342 (1:1000; Invitrogen). For BrdU detection, the sections were pre-treated with 2N-HCl for 30 min at 37°C.

### Experimental procedures

To examine bone-marrow-derived cell (BMDC) infiltration into the LF under consecutive mechanical stress, 1 × 10^7^ bone-marrow cells prepared from CAG-EGFP mice by flushing the femurs and tibias were transplanted into irradiated wild-type recipient mice as previously described [17]. After confirming the reconstitution of >90% EGFP-bone-marrow cells in the chimeric mice, we applied 12-week mechanical stress.

To quantify the number of proliferating cells by consecutive mechanical stress, BrdU (100 μg/g body weight) was intraperitoneally administered daily during the loading period in the two groups.

To induce macrophage infiltration, after peeling the lumbar paraspinal muscles and exposing the mouse LF, we applied micro-injury to the LF at the dorsal side with a sharp 30-gauge needle tip. At 1, 2, and 6 weeks after micro-injury, histological and gene expression analyses were performed. Sham surgery was performed on the controls.

### Human LF samples

Human LF was obtained at surgery from 20 LSCS patients (mean age 68.6 years, range 65–78 years, as hypertrophied LF) and 10 lumbar disc herniation (LDH) patients (mean age 29.9 years, range 24–34 years, as non-hypertrophied LF). Hypertrophied LF from LSCS patients was then divided into two groups: mild (<4 mm) and severe (>4 mm) hypertrophy (n = 10 per group).Human LF sections were subjected to HE and EVG staining. All procedures were approved by the Kyushu University Institutional Review Board (25–126) and the analyses of the sample were performed after obtaining written informed consent from each patient.

### Image acquisition and quantification

All radiographs of the mouse spine were scanned with micro-computed tomography (CT) (60 kV, 50 μm per pixel, Rigaku, Tokyo, Japan). For histological and immunohistochemical analyses, images were obtained using a BZ-9000 digital microscope (Keyence, Osaka, Japan). The thickness of human LF was measured at the facet joint level on axial T1-weighed magnetic resonance imaging (MRI) as previously described [18].The thickness of the mouse LF with/without mechanical stress (the mechanical stress group and the control group) was also measured at the facet joint level on the axial sections with EVG staining (n = 5 per group). To calculate the area of collagen and elastic fibers after EVG staining, we used the ImageJ software program (National Institutes of Health). The area was calculated 3 times and the average value was taken [5]. The number of LF cells in human samples was counted by HE staining of the sagittal sections in 5 random fields at 400× magnification as previously described (n = 10 per group) [19]. The number of LF cells, BMDCs, and proliferating cells in our mouse model was quantified by counting Hoechst, EGFP, and BrdU-immnunopositive cells in the axial sections at the L5-L6 facet joint level at 200× magnification (n = 5 per group). The algorithms for counting the cells were provided by the measurement software program Dynamic cell count BZ-H1c (Keyence).

### Quantitative reverse transcription-polymerase chain reaction (qRT-PCR) Analysis

Total RNA was isolated from LF cells using the RNeasy Mini Kit (Qiagen, Hilden, Germany) and cDNA was synthesized from the total RNA using a PrimeScript reverse transcriptase (TaKaRa, Shiga, Japan) according to the manufacturer’s instructions. Quantitative RT-PCR was performed using 20 μl reaction mixture with primers specific to the genes of interest (Table 1) and SYBR Premix Dimmer-Eraser (TaKaRa) [20]. The mRNA levels in each sample were normalized to those of glyceraldehyde-3-phosphate dehydrogenase mRNA (n = 5 per group).

**Table 1:** Primers used for quantitative reverse transcription polymerase chain reaction.

Gene (Accession Number)		5'- Primer -3'
COL1A1 (NM_007742.3)	Forward	AACCCTGGAAACAGACGAACAACC
	Reverse	TGGTCACGTTCAGTTGGTCAAAGG
COL1A2 (NM_007743.2)	Forward	ATCCAACTAAGTCTCCTCCCTTGG
	Reverse	CTCTGTGGAAGATAGTCAGAAGCC
COL3A1 (NM_009930.2)	Forward	TAAAGAAGTCTCTGAAGCTGATGG
	Reverse	ATCTATGATGGGTAGTCTCATTGC
TNF-α (NM_013693.2)	Forward	TTATGGCTCAGGGTCCAACTCTGT
	Reverse	TGGACATTCGAGGCTCCAGTGAAT
IL-1β (NM_013693.2)	Forward	GGGCTGGACTGTTTCTAATGCCTT
	Reverse	CCATCAGAGGCAAGGAGGAAAACA
IL-6 (NM_013693.2)	Forward	GCTCTCCTAACAGATAAGCTGGAG
	Reverse	CCACAGTGAGGAATGTCCACAAAC
CTGF (NM_010217.2)	Forward	GGCCATACAAGTAGTCTGTCAACC
	Reverse	CACTCCAAAAAGTAGGCACACTGC
PDGF-A (NM_008808.3)	Forward	AGACAGATGTGAGGTGAGATGAGC
	Reverse	ACGGAGGAGAACAAAGACCGCACG
TGF-β1 (NM_011577.1)	Forward	TGGACACACAGTACAGCAAGGTCC
	Reverse	ATCATGTTGGACAACTGCTCCACC
VEGF-A (NM_001025257.3)	Forward	CGGAGGCAGAGAAAAGAGAAAGTG
	Reverse	GGGAGAGAGAGATTGGAAACACAG
MMP-2 (NM_008610.2)	Forward	CCTGGTGACTTCAGATTTAAGAGG
	Reverse	GATGTTGAAGAACCAGAAGAGTGG
MMP-9 (NM_013599.3)	Forward	CTGGTGATCTCTTCTAGAGACTGG
	Reverse	ATGCATCTGCAACTACAGATAAGC
GAPDH (NM_004503)	Forward	GACTTCAACAGCAACTCCCACTCT
	Reverse	GGTTTCTTACTCCTTGGAGGCCAT

COL1A1 indicates collagen type 1 alpha 1; COL1A2, collagen type 1 alpha 2; COL3A1, collagen type 3 alpha 1; TNF-α, tumor necrosis factor-α; IL-1β, interleukin-1β; IL-6, interleukin-6; CTGF, connective tissue growth factor; PDGF-A, platelet-derived growth factor-A; TGF-β1, transforming growth factor-β1; VEGF-A, vascular endothelial cell growth factor-A; MMP-2, matrix metalloproteinase-2; MMP-9, matrix metalloproteinase-9; GAPDH, glyceraldehyde-3-phosphate dehydrogenase.

### Statistical analysis

Wilcoxon’s rank sum test was used to compare the medians of the data between two groups for the area, width, and thickness of the LF, the area of the collagen and elastic fibers, the qRT-PCR results, and the cell count in the mouse LF. To analyze the differences among three groups in the ratio of elastic fibers to collagen fibers and the cell count in human LF, ANOVA with the Tukey-Kramer post hoc test was performed. Statistical significance was set at *p* < 0.05. The data were presented as the mean ± SEM. All statistical analyses were carried out using the JMP software program (version 11; SAS Institute, Cary, NC, USA).

## Results

### Consecutive mechanical stress loading to the mouse LF

**Fig 1:**
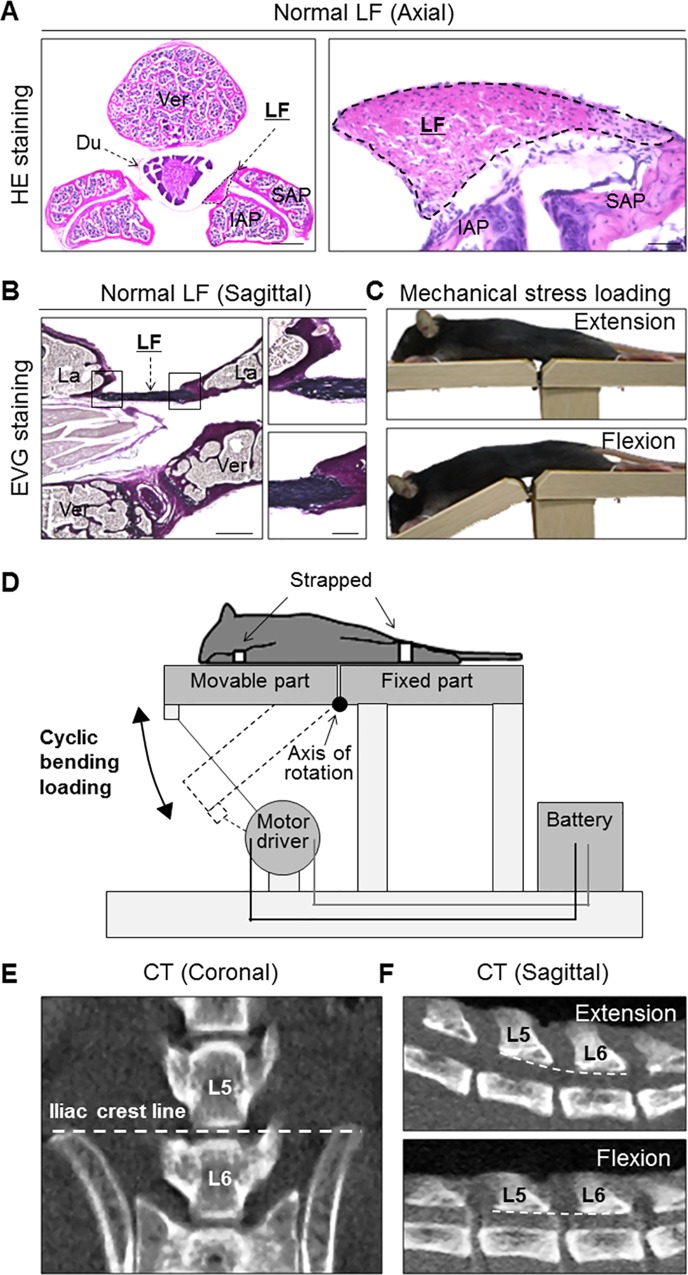
Histological analyses of the mouse lumbar spine and a mechanical stress loading device. The application of mechanical stress to the mouse LF using a novel device. (A) HE and (B) EVG staining of the spine sections. (C) Photographs and (D) schematic illustrations of the device. (E) A coronal CT image showing that the iliac crest line corresponds to the L5-L6 disc level. (F) Sagittal CT images showing that mechanical stress was consistently applied at the L5-L6 level. Scale bars (A): 300 μm; insets: 50 μm; (B): 300 μm; insets: 100 μm. LF, ligamentum flavum; Ver, vertebral body; Du, dural tube; SAP, superior articular process; IAP, inferior articular process; La, lamina.

First, to evaluate whether the mouse was a feasible experimental animal model for studying LF hypertrophy, we performed histological analyses of the mice lumbar spine. In the axial sections of the spine, HE staining showed that the mouse LF was located between the dural tube and facet joints (Fig 1A). In the sagittal sections, EVG staining demonstrated that the LF ran between adjacent laminas and mostly consisted of black-staining elastic fibers (Fig 1B). These histological features were very similar to those of human LF. Therefore, we decided to use mice to examine the pathomechanism underlying LF hypertrophy.

To establish a LF hypertrophy mouse model, we initially developed a loading device by which the mouse LF was subjected to consecutive mechanical stress for the present experiment (Fig 1C). The device consisted of a moving bed, straps, and motor driver (UNIQUE MEDICAL, Tokyo, Japan) (Fig 1D). During the loading, the limbs were firmly strapped onto the bed under anesthesia. When the upper half of the bed was constantly moving, the spine was bent and extended repeatedly at the rate of 20 cycles per minute by the motor. To determine the appropriate loading, we performed a preliminary experiment with 12 weeks of mechanical stress loading for 1.5, 3, and 4.5 h/day, and then decided on a 12-week loading period because an experimental period exceeding 12 weeks was not practical. During this period, some mice in the 4.5 h/day group showed weight and hair loss, whereas no adverse events were noted in the other groups. We therefore performed 3 h/day loading in subsequent experiments. The controls were under anesthesia alone. To apply mechanical stress consistently at the L5-L6 level, we used the iliac crest line as an anatomical land mark of the level (Fig 1E) and confirmed that mechanical stress was adequately loaded to the mouse LF at the level by CT images (Fig 1F).

### Mechanical stress brought about LF hypertrophy

**Fig 2:**
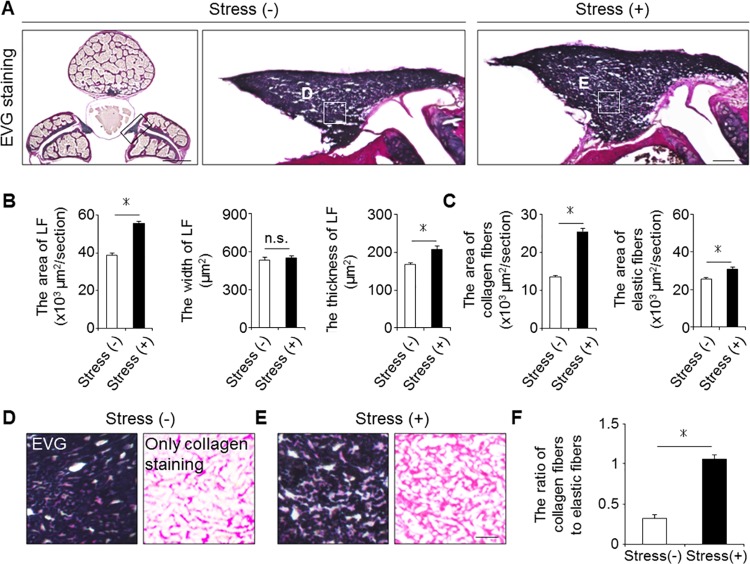
Mechanical stress induced LF hypertrophy. (A) Axial sections of the mouse LF with/without 12-week mechanical stress loading on EVG staining. (B and C) Bar graphs showing the cross-sectional area, width, thickness, and the area of collagen fibers and elastic fibers in the two groups. (D and E) High magnifications of (A). (F) Bar graph showing the ratio of collagen fibers to elastic fibers in the two groups. Scale bars (A): 500 μm; insets: 50 μm; (D and E): 10 μm. *p < 0.05, Wilcoxon’s rank sum test, n.s. = not significant (n = 5/group).

To examine whether mechanical stress indeed induced LF hypertrophy, we compared the axial cross-sectional area of the mouse LF with/without mechanical stress loading (the stress group vs. the control group, respectively). We found the sectional area in the stress group to be about 1.5-fold that in the control group on EVG staining after 12-week mechanical stress (Fig 2A and 2B). Although the width was comparable between the two groups, the thickness of the stress group was significantly higher than that of the control group (Fig 2B). These results indicated the successful establishment of a LF hypertrophy mouse model via mechanical stress.

We then investigated the effect of mechanical stress on the extracellular matrix (ECM). Previous observations in human samples demonstrated that the major ECM component was elastic fibers, while the minor component was collagen fibers in non-hypertrophied LF, whereas the ratio of collagen fibers was markedly increased in hypertrophied LF [4,5]. Similarly, in our mouse model, the area of collagen fibers was considerably higher in the stress group than in the control group (Fig 2C). Although the area of elastic fibers also increased, the density of elastic fibers decreased compared to that of collagen fibers (Fig 2C–2F).

### The histological comparison of the mouse and human LF

**Fig 3:**
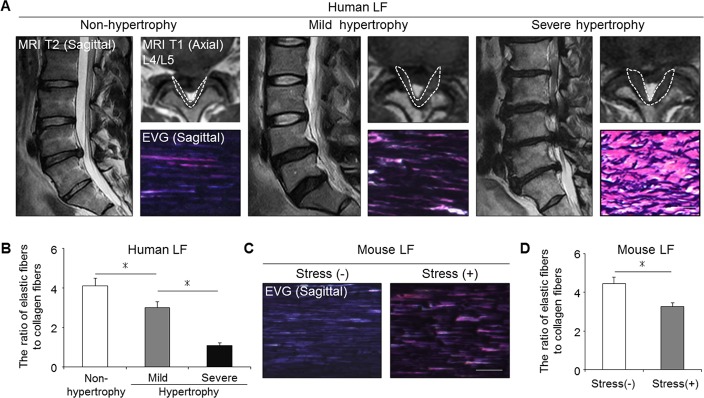
Our mouse model histologically reflected mildly hypertrophied LF in humans. (A) MRI and EVG staining of human samples: non-hypertrophy and mild and severe hypertrophy. The white broken lines indicate outlines of the LF. (B) Bar graph showing the ratio of elastic fibers to collagen fibers in the three groups. *p < 0.05, an ANOVA with the Tukey-Kramer post hoc test (n = 10/group). (C) EVG staining of the mouse LF with/without 12-week mechanical stress. (D) Bar graph showing the ratio of elastic fibers to collagen fibers in the two groups. *p < 0.05, Wilcoxon’s rank sum test (n = 5/group). Scale bars (A): 100 μm; (C): 50 μm.

We next evaluated the severity of LF hypertrophy in the mouse model in comparison to human samples. Non-hypertrophied LF of human showed a dense and regular bundle of elastic fibers, whereas severely hypertrophied LF showed thin, irregular, and fragmented elastic fibers in EVG staining (Fig 3A). Additionally, the elastin-to-collagen ratio decreased in hypertrophied LF compared to non-hypertrophied LF (Fig 3B). In our mouse model, the elastic fibers in the control group were dense and aligned, whereas in the stress group, they were slightly degenerated, and a decreased elastin-to-collagen ratio was observed (Fig 3C and 3D). These results indicated that our mouse model by mechanical stress was histologically identical to mildly hypertrophied LF of human.

### Increased number of LF cells by mechanical stress

**Fig 4:**
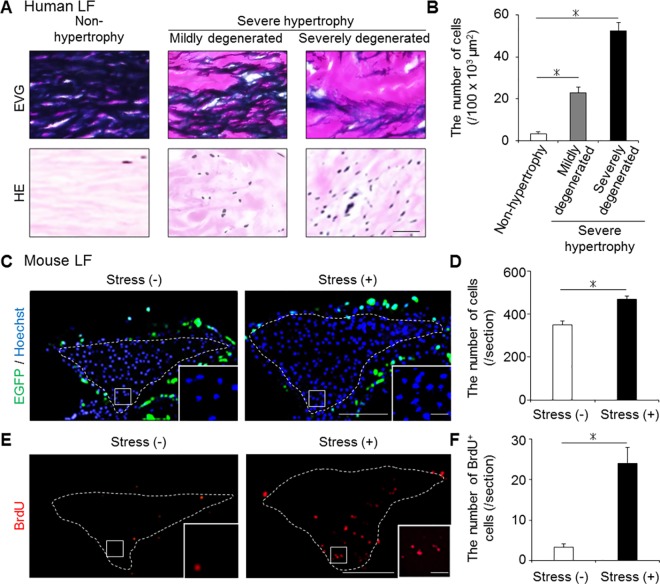
The changes in the cellular distribution in the hypertrophied LF of mice and humans. (A and B) The number of cells significantly increased with LF hypertrophy in humans. *p < 0.05, an ANOVA with the Tukey-Kramer post hoc test (n = 10/group). (C-F) The comparison of the number of cells (Hoechst, blue), infiltrating BMDCs (EGFP, green), and proliferating cells (BrdU, red) in the mouse LF with/without 12-week mechanical stress. To detect BMDCs, we generated bone-marrow-chimeric mice by transplanting from CAG-EGFP mice. The white broken lines indicate the outlines of the LF. *p < 0.05, Wilcoxon’s rank sum test (n = 5/group). Scale bars (A): 100 μm; (C and E): 100 μm; insets: 10 μm.

In addition to these ECM component changes, we examined the cellular distribution changes using human and our mouse samples. There were few cells in human non-hypertrophied LF, whereas a significantly increased number of cells was observed in human hypertrophied LF (Fig 4A and 4B). Also in our mouse model, the number of Hoechst-positive LF cells was significantly higher in the stress group than in the control group (Fig 4C and 4D). Furthermore, the number of BrdU-positive proliferating cells was significantly higher in the stress group (Fig 4E and 4F). We initially expected to observe BMDC infiltration by mechanical stress because macrophage infiltration was reported in human hypertrophied LF [21]. However, no EGFP-positive BMDC infiltration occurred in the bone-marrow-chimeric mouse LF with/without mechanical stress (Fig 4C).

### Increased gene expression of fibrosis-related factors by mechanical stress

**Fig 5:**
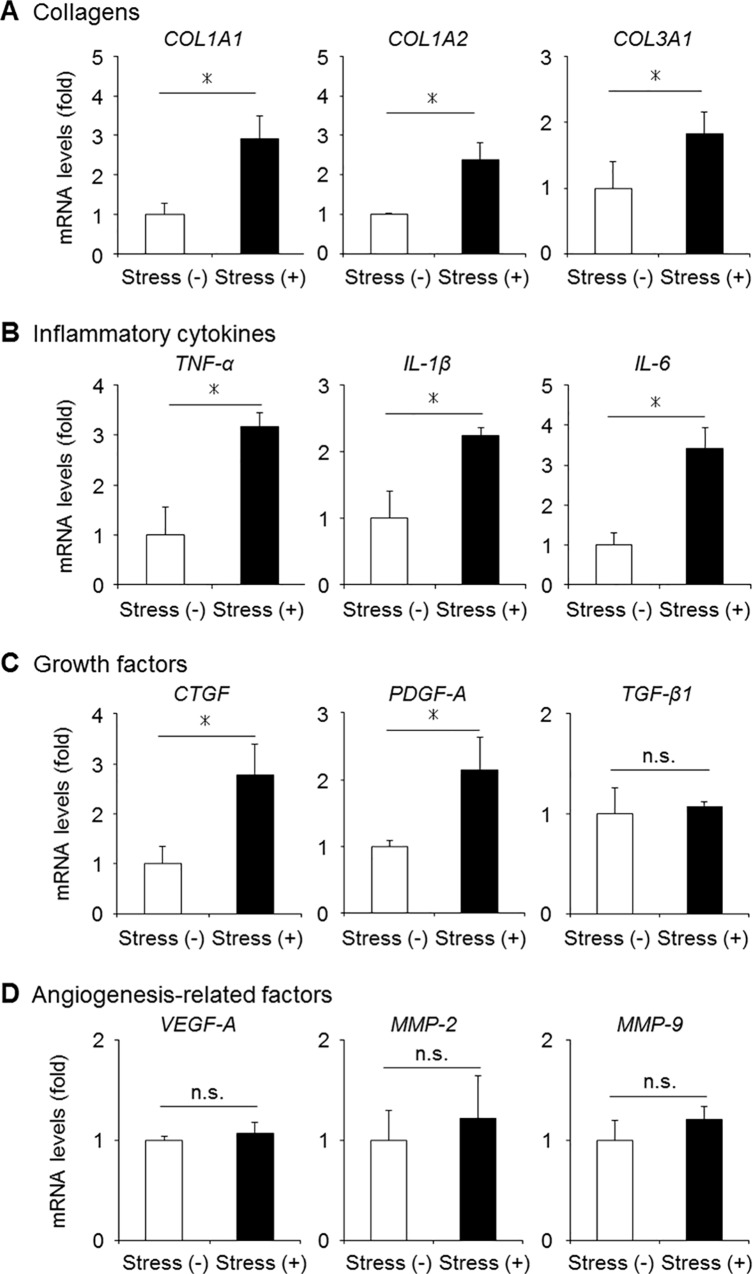
The mRNA expression of fibrosis-related factors in our mechanical stress mouse model. (A-D) The gene expression of collagens, inflammatory cytokines, growth factors, and angiogenesis-related factors evaluated in the mouse LF with/without mechanical stress loading by qRT-PCR (n = 5/group). *p < 0.05, Wilcoxon’s rank sum test, n.s. = not significant. COL1A1, collagen type 1 alpha 1; TNF-α, tumor necrosis factor-α; IL, interleukin; CTGF, connective tissue growth factor; PDGF-A, platelet-derived growth factor-A; TGF-β1, transforming growth factor-β1; VEGF-A, vascular endothelial cell growth factor-A; MMP, matrix metalloproteinase.

To examine the influence of mechanical stress on the activation of fibrosis-related factors in LF cells, we evaluated the gene expression of inflammatory cytokines, growth factors, and angiogenesis-related factors in the mouse model. Quantitative RT-PCR demonstrated that the gene expression of collagens, tumor necrosis factor-α, interleukin-1β, and interleukin-6 were significantly higher in the stress group than in the control group (Fig 5A and 5B). A significant increase in the CTGF and PDGF-A expression was also observed in the stress group (Fig 5C). Although an abundant TGF-β1 expression and angiogenic factors were reported in severely hypertrophied LF of humans [21], no significant differences were seen in the two mouse groups (Fig 5C and 5D).

### Micro-injury-induced macrophage infiltration and collagen accumulation in the injured area

**Fig 6:**
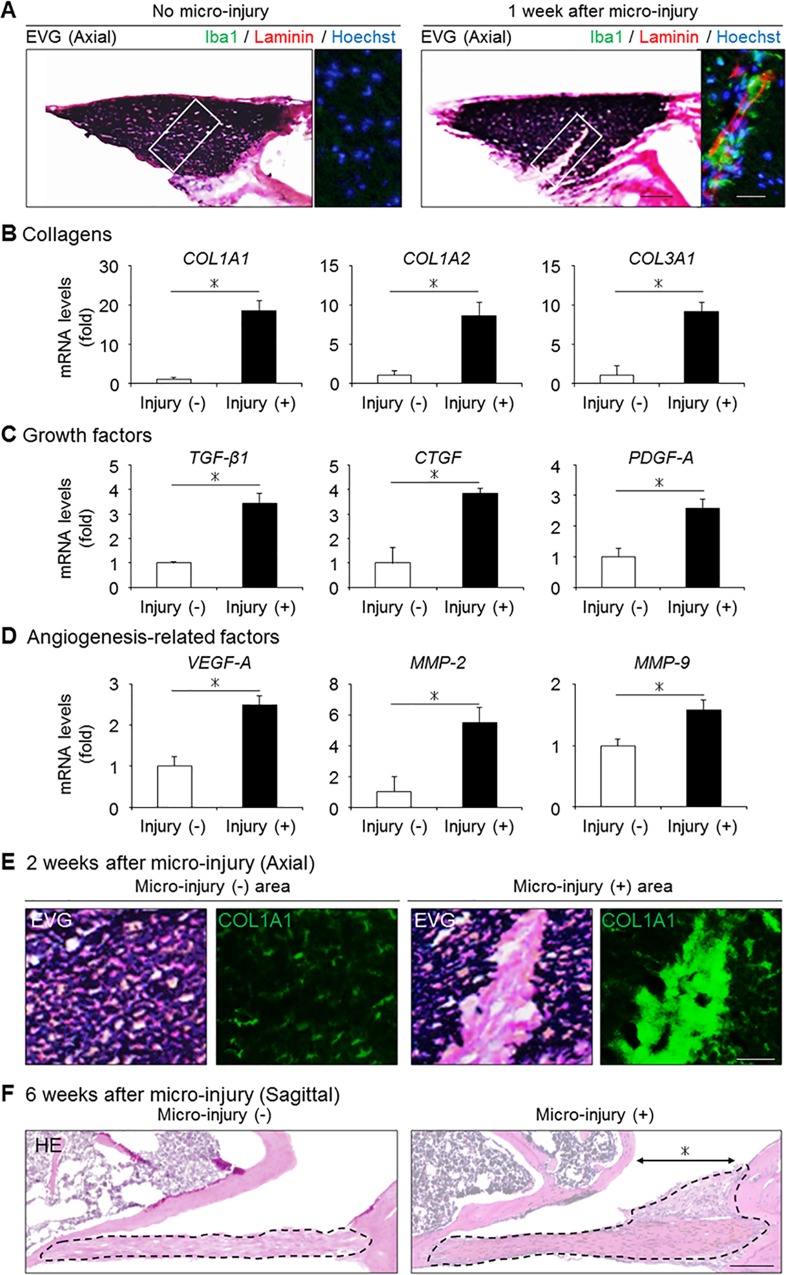
The influence of macrophage infiltration following micro-injury on the mouse LF. (A) The presence of infiltrating macrophages (Iba1, green) and neovascular vessels (laminin, red) in the mouse LF with/without micro-injury. (B-D) Bar graphs showing the gene expression of collagens, fibrosis-related growth factors, and angiogenesis-related factors in the two groups. (E) The collagen synthesis in the injured area (COL1A1, green) at 2 weeks after micro-injury. (F) Sagittal sections of the mouse LF with/without micro-injury on HE staining at 6 weeks after micro-injury. The black broken lines indicate the outlines of the LF. The asterisk indicates the area to which micro-injury was applied. *p < 0.05, Wilcoxon’s rank sum test (n = 5/group). Scale bars (A): 50 μm; insets: 20 μm; (E): 20 μm; (F) 200 μm. COL1A1, collagen type 1 alpha 1; TGF-β1, transforming growth factor-β1; CTGF, connective tissue growth factor; PDGF-A, platelet-derived growth factor-A; VEGF-A, vascular endothelial cell growth factor-A; MMP, matrix metalloproteinase.

In contrast to severely hypertrophied LF of LSCS patients, our mouse model by mechanical stress demonstrated no infiltrating macrophages, increase in TGF-β1 expression, or angiogenesis (Figs 4C, 5C and 5D). We therefore hypothesized that factors other than mechanical stress were involved in the progression of LF hypertrophy. To examine the pathological role of macrophages in LF hypertrophy, we induced macrophage infiltration by applying micro-injury to the normal mouse LF at the dorsal side. At 1 week after micro-injury, Iba1-positive macrophages had infiltrated around the injured lesion (Fig 6A). Notably, a qRT-PCR analysis revealed the gene expression of collagens and fibrosis-related growth factors, including TGF-β1, to be significantly higher in the micro-injured group than in the non-injured group (Fig 6B and 6C). Furthermore, laminin-positive micro-vessels and a significant increase in the levels of angiogenesis-related factors were also observed (Fig 6A and 6D). Indeed, excessive collagen synthesis without elastic fibers was observed in the injured area at 2 weeks after micro-injury (Fig 6E), and LF hypertrophy was detected selectively in the micro-injury area at 6 weeks after injury (Fig 6F). These results strongly suggested that macrophage infiltration was a significant factor involved in the progression of LF hypertrophy by activating collagen production.

## Discussion

In this study, by applying consecutive mechanical bending stress to the mouse LF, we demonstrated that mechanical stress was one of the direct causes of LF hypertrophy. In the mouse hypertrophied LF, increased collagen fibers, proliferating cells, and the gene expression of several fibrosis-related factors were found. In addition, macrophage infiltration with angiogenesis was induced by applying micro-injury to the mouse LF. In this macrophage infiltration model, LF hypertrophy was observed along with the excessive expression of collagen and the increased expression of TGF-β1. These findings suggest that long-term mechanical stress and macrophage infiltration significantly influence the progression of LF hypertrophy.

To date, previous studies have reported the common histological characteristics of human hypertrophied LF as follows: collagen deposition, elastic fiber fragmentation, and calcification [22,23]; inflammatory cell accumulation [21]; and an increased expression of fibrosis-related factors [6–8,24,25]. However, these pathological changes in human samples only indicated the advantaged stage of LF hypertrophy, and determining which factors contribute to the process of LF hypertrophy is difficult. Therefore, we established an experimental animal model to clarify its pathomechanisms. In our mechanical stress model, we confirmed the increases in collagen fibers, cell proliferation, and the fibrosis-related factors expression, in line with human LF pathology.

In fibrotic diseases of several organs, TGF-β1 has been reported to be an important disease-related factor for collagen production [26,27]. The main source of this cytokine was believed to be infiltrating macrophages in fibrosis [28,29]. For example, the number of infiltrating macrophages correlated with the TGF-β1 expression and the progression of collagen accumulation in liver fibrosis [28]. In addition, in severely hypertrophied LF of humans, macrophage infiltration was observed in the collagen deposition area with increased TGF-β1 expression [21]. In the development of a LF hypertrophy mouse model, we hypothesized that consecutive mechanical stress would induce macrophage infiltration and increased TGF-β1 expression. However, although the increased gene expression of several fibrosis-related factors was observed in our mechanical stress mouse model, there was no BMDC infiltration or increased TGF-β1 expression (Figs 4C and 5C). Therefore, we additionally induced macrophage infiltration by applying micro-injury, and found that macrophages were associated with the increased expression of collagens and TGF-β1 (Fig 6A–6C). Indeed, we found excessive collagen synthesis in the injured site at 2 weeks after micro-injury (Fig 6E) and confirmed LF hypertrophy at 6 weeks after micro-injury (Fig 6F). These results suggested that infiltrating macrophages may also play a significant role in the progression of LF hypertrophy via the increased expression of TGF-β1.

Another characteristic of human hypertrophied LF is angiogenesis. While the normal LF is non-vascularized, marked angiogenesis was observed in the area of collagen accumulation in the severely hypertrophied LF [21]. Several factors such as VEGF and MMPs are considered to be important for angiogenesis, and their actual expression has been mainly observed in fibrotic areas [30]. These angiogenic factors were reported to be derived from infiltrating macrophages, and neovascular vessels further promoted macrophage infiltration, resulting in the excessive expression of TGF-β1 as well as collagen production [31]. Therefore, the interplay between infiltrating macrophages and angiogenesis may also worsen the fibrotic pathology in LF hypertrophy progression. In our macrophage infiltration model, angiogenesis and the significantly increased expression of angiogenic factors were observed with infiltrating macrophages (Fig 6A and 6D). This macrophage infiltration model may help elucidate the effect of disrupting the cycle of macrophage infiltration and angiogenesis to prevent the progression to severe LF hypertrophy. Indeed, an angiogenesis inhibitor was reported to successfully suppress macrophage infiltration and subsequently prevent fibrotic collagen accumulation in renal fibrosis [32].

There are several limitations associated with the present study. We did not establish a LF hypertrophy mouse model showing advanced histological changes only by mechanical stress. Although we tried to investigate the influence of the combination of mechanical stress and micro-injury on LF hypertrophy, the combination unexpectedly caused an LF tear and the sample could not be analyzed. In addition to this limitation, our mechanical stress mouse model cannot be used to develop an LSCS model because the ratio of the LF to the dural tube was significantly smaller in mice than in humans. Nevertheless, we believe that each model we established in this study showed the pathological characteristics of human hypertrophied LF, at least in part, and is thus useful for a better understanding its pathogenesis.

In conclusion, we demonstrated for the first time that consecutive mechanical stress directly brought about LF hypertrophy in a mouse model. In addition, macrophage infiltration following micro-injury was found to be associated with severe LF hypertrophy by stimulating angiogenesis, collagen synthesis, and increased TGF-β1 production.
